# A Compact Mid-Infrared Spectroscopy System for Healthcare Applications Based on a Wavelength-Swept, Pulsed Quantum Cascade Laser

**DOI:** 10.3390/s20123438

**Published:** 2020-06-18

**Authors:** Takuya Koyama, Naoto Shibata, Saiko Kino, Atsushi Sugiyama, Naota Akikusa, Yuji Matsuura

**Affiliations:** 1 Graduate School of Biomedical Engineering, Tohoku University, Sendai 908-8579, Japan; takuya.koyama.r2@dc.tohoku.ac.jp (T.K.); naoto.shibata.93@gmail.com (N.S.); kino@ecei.tohoku.ac.jp (S.K.); 2 Hamamatsu Photonics K.K., Hamamatsu 434-8601, Japan; a-sugi@cfc.hpk.co.jp (A.S.); aki@lpd.hpk.co.jp (N.A.)

**Keywords:** quantum cascade laser, mid-infrared spectroscopy, ATR spectroscopy, hollow optical fiber

## Abstract

A mid-infrared spectroscopic system using a high-speed wavelength-swept and pulsed quantum cascade laser (QCL) for healthcare applications such as blood glucose measurement is proposed. We developed an attenuated total reflection measurement system comprising the QCL with a micro-electromechanical system (MEMS)-scanning grating, hollow optical fibers, and InAsSb detector and tested its feasibility for healthcare applications. A continuous spectrum was obtained by integrating comb-shaped spectra, the timing of which was slightly shifted. As this method does not require complex calculations, absorption spectra are obtained in real-time. We found that the signal-to-noise ratio of the obtained spectrum had been improved by increasing the number of spectra that were integrated into the spectrum calculation. Accordingly, we succeeded in measuring the absorption spectrum of a 0.1% aqueous glucose solution. Furthermore, the absorption spectra of human lips were measured, and it was shown that estimation of blood glucose levels were possible using a model equation derived using a partial least squares regression analysis of the measured absorption spectra. The spectroscopic system based on the QCL with MEMS-scanning grating has the advantages of compactness and low cost over conventional Fourier transform infrared-based systems and common spectroscopic systems with a tunable QCL that has a relatively large, movable grating.

## 1. Introduction

In recent years, research on biomedical sensing using mid-infrared light with a wavelength of 3 to 12 μm has been actively conducted by a variety of groups [1,2]. In this wavelength region, sharp and strong absorption peaks appear because of the fundamental vibrations of various molecules that make up biological tissue. Accordingly, biological tissue can be analyzed by investigating the absorption spectrum (the so-called “fingerprint spectrum”) formed by these molecules, making it possible to identify the constituent molecules and analyze their components. In principle, by using mid-infrared absorption spectroscopy, components such as proteins, sugars, and lipids contained in blood or interstitial fluid can be analyzed non-invasively. Therefore, the development of a healthcare system performing health checkups on-site and in real-time without blood sampling is anticipated.

Although a mid-infrared spectroscopic system based on a Fourier transform infrared spectrophotometer (FT-IR) has generally been used for biomedical applications, the FT-IR has an interferometer with a scanning system, which makes it difficult to downsize the system. Furthermore, since FT-IR uses an incoherent lamp source such as a Globar, which emits relatively low optical power, a highly sensitive HgCdTe detector cooled by liquid nitrogen is required to obtain a sufficient signal-to-noise ratio (SNR). This has also been a problem for the development of a compact healthcare system.

Contrastingly, mid-infrared quantum cascade lasers (QCLs), which have been developed in recent years, can generate a high optical output power of 100 mW or more from a small laser chip [3,4]. A compact system can thus be realized by combining the QCL with a semiconductor detector operating at room temperature. An InAsSb detector presents the most promising option in this context because it does not use regulated materials included in the Restriction of Hazardous Substances Directive (RoHS) such as Hg and Cd [5] and is already commercially available.

Furthermore, by combining a flexible optical fiber transmitting mid-infrared light with these light sources and detectors, it is possible to easily construct a portable spectroscopic system suitable for healthcare applications. Although chalcogenide glass optical fiber and polycrystalline optical fiber are commercially available as types of mid-infrared optical fiber, the latter is advantageous because it does not contain toxic material prohibited from being used in biomedical applications. Various proposals for spectroscopic systems using a QCL and AgCl/AgBr polycrystalline optical fiber have been made [6,7]. Moreover, because of its simpler structure, a hollow optical fiber is advantageous in terms of cost and durability as an optical transmission medium for mid-infrared spectroscopy systems. Hollow optical fiber is a flexible, thin glass or plastic tube in which metal and dielectric thin films are formed [8]. By setting the dielectric film height appropriately, low-loss transmission in the targeted wavelength band in the mid-infrared region [9] can be realized. A spectroscopic system combining the hollow optical fiber with QCL was also proposed [10,11,12].

The QCL-based systems have already been applied to various biomedical applications [13] including the detection of NO gas [14], ammonia detection [15] in breath, blood glucose concentration measurement [7,16,17,18], and drug detection in saliva [19]. For these application systems, an external cavity-type QCL (EC-QCL) capable of wavelength sweeping is typically used. Since continuous spectra in the mid-infrared region are acquired by using EC-QCL, it can be used as a better alternative to conventional FT-IR. However, EC-QCL with a movable grating presents some problems in terms of size and cost, and it is currently difficult for use in a compact and inexpensive healthcare system. On the contrary, we attempted to measure blood glucose level by a system using multiple distributed feedback (DFB) QCLs with a single wavelength. The DFB-QCL-based system can be miniaturized because it does not require a movable resonator. Furthermore, some continuous-wave oscillating devices packaged in a small CAN package are currently available on the market. We extracted a small number of discrete wavelengths that showed a high correlation with the blood glucose concentration from the absorption spectrum of the human lip in the mid-infrared region, obtained by the attenuated total reflection (ATR) method using FT-IR [18,20,21,22]. However, to date, the results obtained using only discrete wavelengths have been slightly inferior in accuracy to blood glucose measurements using a continuous spectrum [18].

A high-speed wavelength-swept, pulsed QCL may present a solution to the shortcomings of EC-QCL and DFB-QCL. This QCL uses high-speed operating micro-electromechanical systems (MEMS) grating as an external resonator [23]. By combining it with a pulsed QCL, which is advantageous in terms of cooling, downsizing is possible and the future cost of such a system can be kept low. In this paper, we report the results of measuring the absorption spectrum of lip mucosa by constructing a compact ATR measurement system that combines this high-speed wavelength-swept, pulsed QCL, a hollow optical fiber for mid-infrared transmission, and an InAsSb detector. This paper consists of four sections. After this Introduction, the experimental setup used in this work is reported. In Section 2, a continuous spectrum was obtained by integrating comb-shaped spectra resulting from the pulse train emitted from the QCL in the data processing of measured data. As this method does not require complex calculations, absorption spectra are obtained in real-time. In the following Section 3, we firstly measured the absorption spectra of aqueous glucose solution to evaluate the sensitivity. Then the absorption spectra of human lips were measured and partial least squares regression analysis was applied to the measured absorption spectra. In Section 4, we discuss these results and we will show the feasibility of the proposed system.

## 2. Experimental Setup

In this research, a wavelength-swept, pulsed QCL (Hamamatsu Photonics, Hamamatsu, Japan, L14890-09) was used as a light source. It uses a broadband gain medium and a MEMS-scanned grating in a compact 80 mm cube package. The wavelength sweep range is roughly 8.5 to 10.3 µm, and the repetition frequency of the MEMS grating is 1.8 kHz. It emits pulses with a peak power higher than 200 mW at a repetition rate of 180 kHz. The MEMS grating and optical pulses are triggered using a multifunction generator (NF corporation, Yokohama, Japan, WF1974). The optical signal from the detector was acquired using an oscilloscope (Keysight Technologies, Santa Rosa, CA, USA, DSOX2014A). The signal was processed an average of 1024 times.

**Figure 1:**
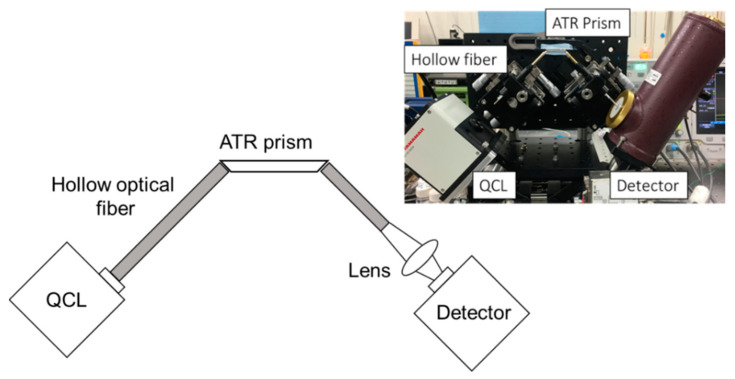
Schematic and appearance of the attenuated total reflection (ATR) measurement system using the quantum cascade laser (QCL).

Figure 1 shows a schematic and the appearance of the ATR measurement system using this QCL. The infrared light emitted from the QCL is collimated and guided to the ATR prism by a hollow optical fiber with an inner diameter of 2 mm and a length of 10 cm. The hollow optical fiber emits almost a parallel beam because of the small numerical aperture (NA < 0.05) of hollow optical fibers. Moreover, the profile of the emitted beam coincides well with Gaussian. Therefore, the use of hollow optical fiber does not affect sensor performance. The prism is made of ZnS crystal that is non-toxic to the human body and transparent in the wavelength range of 10 μm. The ZnS ATR prism has a trapezoid shape and the length is set to 24 mm to be suitable for human lips. The prism thickness of 2.4 mm was chosen so that the output beam from the 2-mm diameter hollow-optical fiber can be entered into the prism. The height of the prism was firstly set to 2.4 mm to occur nine reflections at the top and bottom surfaces of the prism whose end faces are cut at 45°. After the infrared light from the prism is emitted from the hollow optical fiber, the spot diameter is reduced by a ZnSe lens with a focal length of 25 mm and is incident on the detector. Liquid nitrogen-cooled HgCdTe (Hamamatsu Photonics, Yokohama, Japan, P2748-40) and electron-cooled InAsSb (Hamamatsu Photonics, Yokohama, Japan, P13894-211MA) detectors were used.

**Figure 2:**
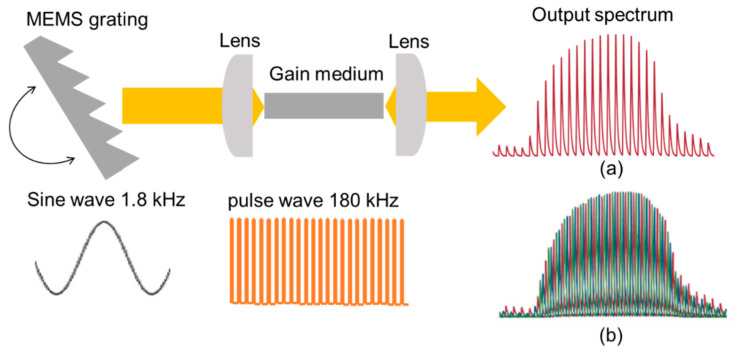
The schematic internal structure of the QCL and examples of output spectrum shape. (**a**) Comb-shaped spectrum shape of a single scan, (**b**) a spectrum shape produced by overlapping comb-shaped spectra with a different phase shift.

**Figure 3:**
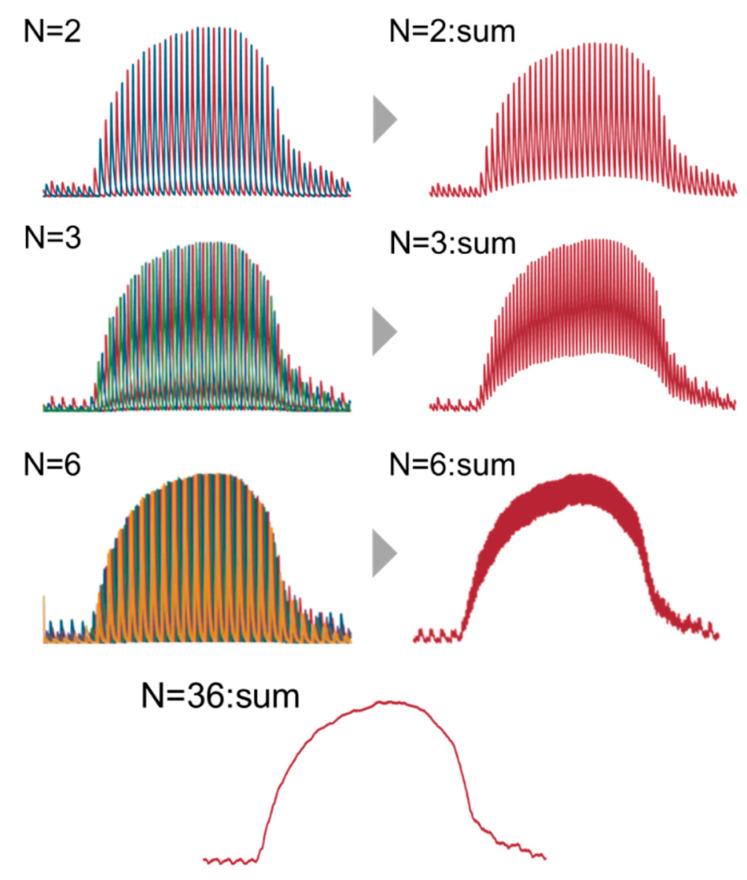
The method for obtaining a continuous spectrum from pulsed spectra.

Figure 2 illustrates the schematic internal structure of the QCL and an example of the output spectrum shape (a). The output light adopted a comb-shaped spectrum because it is the product of the gain spectrum obtained by the grating, driven by a 1.8 kHz sine wave and the 180 kHz pulsed light. The output pulses were arranged at equal frequency intervals on the time axis. Then, by providing a phase shift between the sine and the pulse wave, the frequency timing could be shifted as shown in the spectrum (b). To obtain a continuous spectrum from this spectrum (b), it is common to detect the peak values of each pulse and to connect them in a continuous line. However, this method requires significant calculation, and it is difficult to obtain a spectrum in real-time. Therefore, we attempted to obtain a continuous spectrum by simply adding up the multiple spectra obtained by the phase shift. Since the shape of each pulse has a specific spectrum width, it is possible to obtain a continuous spectrum without spikes by increasing the number of phase shifts and reducing the frequency interval (see Figure 3). We calculated the coefficient of variation of the measured spectrum with a different number of phase shifts. As a result, we found that the small deviation could be obtained when the number of phase shifts was more than 36. Considering the data acquisition time and the signal-to-noise ratio, we set the number of phase shifts as 36 and the time required for spectrum acquisition was 10 s.

This study was approved by the Tohoku University Clinical Research Review Board (certification number: CRB2180001). Written informed consent was obtained from the subject for publication of this paper.

## 3. Results

**Figure 4:**
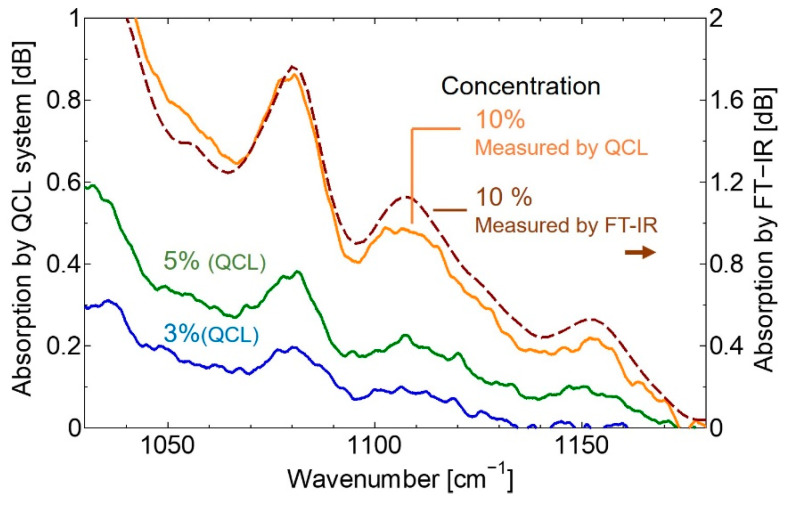
The absorption spectra of the glucose solution.

To investigate the accuracy of the measurement system for biomedical spectroscopic applications, the absorption spectrum of the glucose solution was measured first. To obtain the absorption spectrum, we firstly measured the transmission spectrum of pure water as a reference spectrum. Then the spectrum of the glucose aqueous solution was measured, and the absorption spectrum was calculated by dividing the glucose spectrum by the reference spectrum. Figure 4 shows the obtained absorption spectra. Additionally, the spectrum of a 10% glucose aqueous solution measured using FT-IR is also shown for comparison.

**Figure 5:**
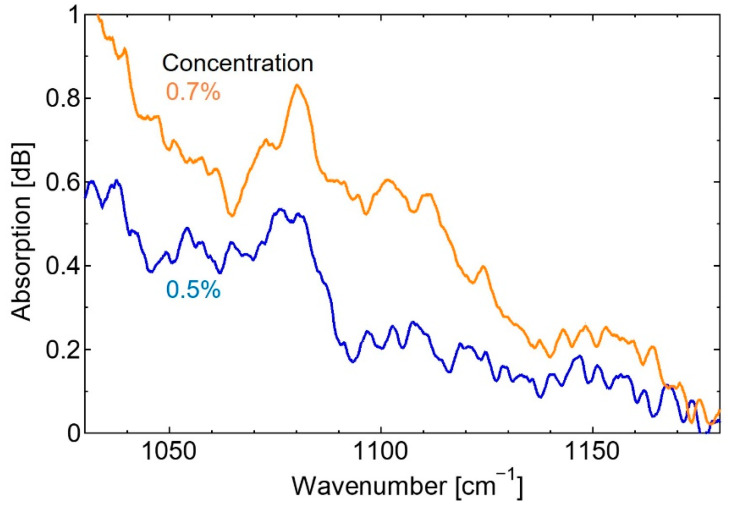
Absorption spectra of the glucose solution after changing the prism.

After this experiment, the height of ATR trapezoidal prism was changed from 2.4 mm to 1.2 mm to improve the measurement sensitivity. Figure 5 shows the measurement results of the glucose aqueous solution after changing the prism.

To further improve the SNR of the measurement system, the number of phase shifts was increased. Accordingly, we adopted a high-speed amplifier combined with an InAsSb detector (Hamamatsu Photonics, Yokohama, Japan, P13894-211MA). The bandwidth of this amplifier was 1 MHz, which is significantly faster than that of the HgCdTe detector (150 kHz bandwidth); this resulted in narrowing the pulse width of each comb-shaped spectrum. Subsequently, a larger number of comb-shaped spectra could be integrated to reduce noise.

**Figure 6:**
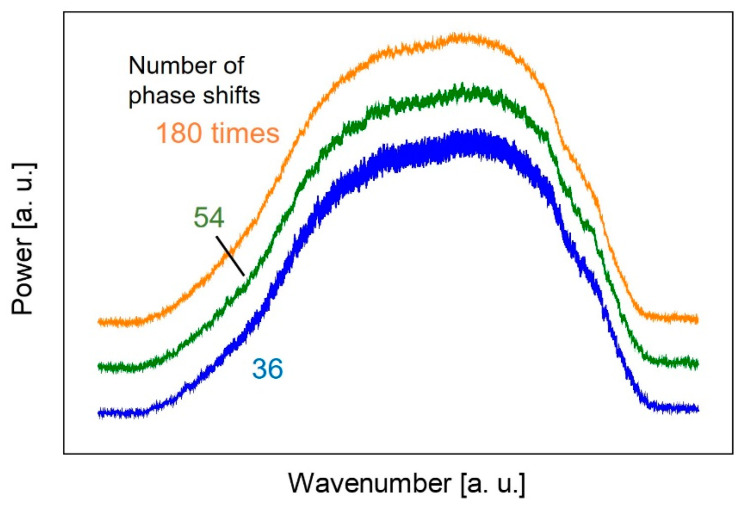
The power spectra measured at three different phase shift numbers.

**Figure 7:**
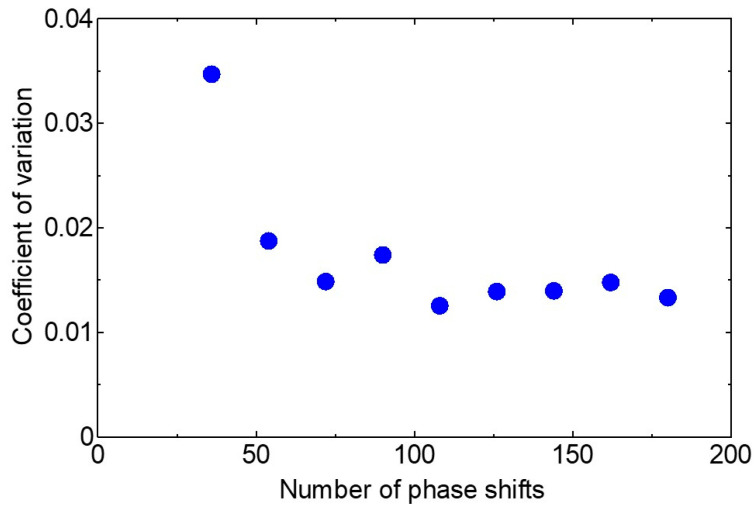
The relationship between the cofficient of variation of spectrum and the number of phase shifts after the improvement of the system.

Figure 6 shows the power spectrum measured at three different phase shift numbers. The noise was visibly reduced by increasing the number of phase shifts. Figure 7 shows the relationship between the coefficient of variation of the spectrum and the number of phase shifts. From this result, we found that a spectrum less affected by noise could be obtained when the number of phase shifts was more than 60.

**Figure 8:**
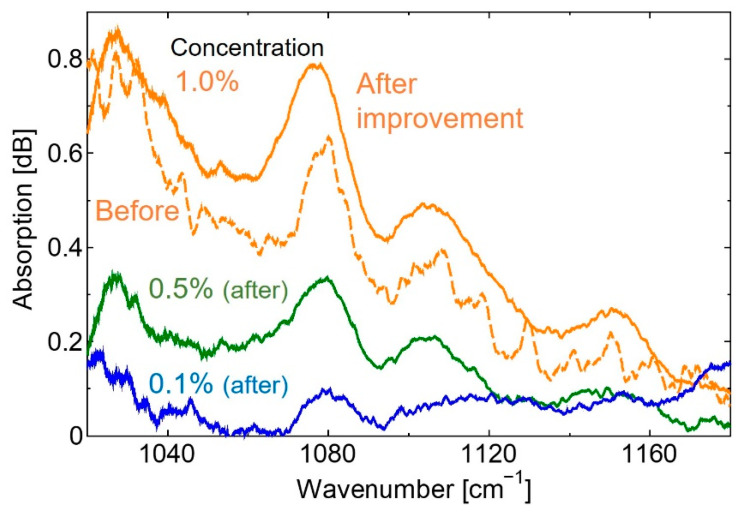
The absorption spectra of the glucose solution provided by the improved system.

Figure 8 shows the absorption spectra of aqueous glucose solutions with different concentrations measured using the improved measurement system. In this figure, an absorption spectrum of 1.0% glucose solution measured by the system with a slower amplifier is also shown for comparison.

**Figure 9:**
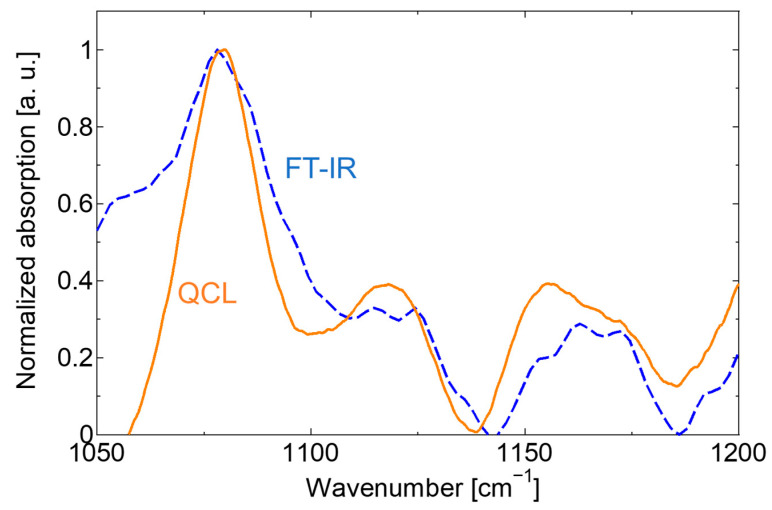
The absorption spectra of the lip mucosa.

Figure 9 shows the absorption spectrum of lip mucosa; the spectrum measured by the FT-IR system is also shown for comparison. In the measurement, the upper and bottom surfaces of the prism were touched by upper and lower lips.

**Figure 10:**
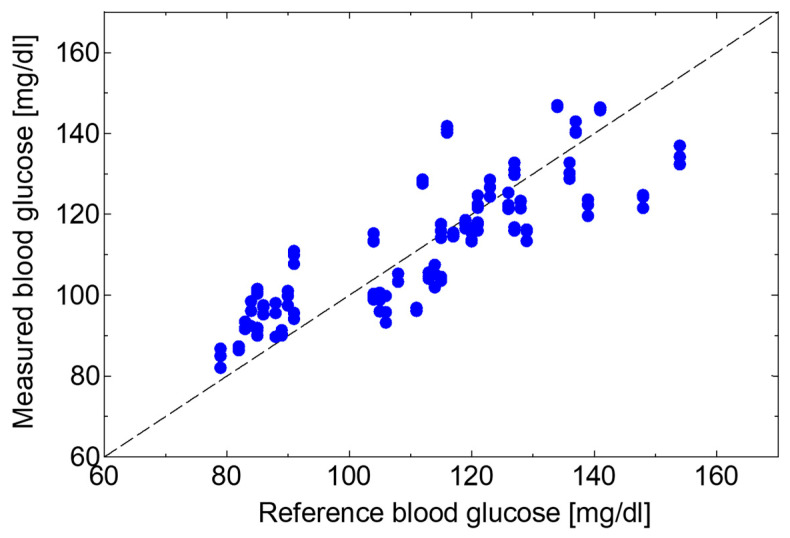
The partial least squares regression (PLSR) analysis result from data over 4 days.

**Figure 11:**
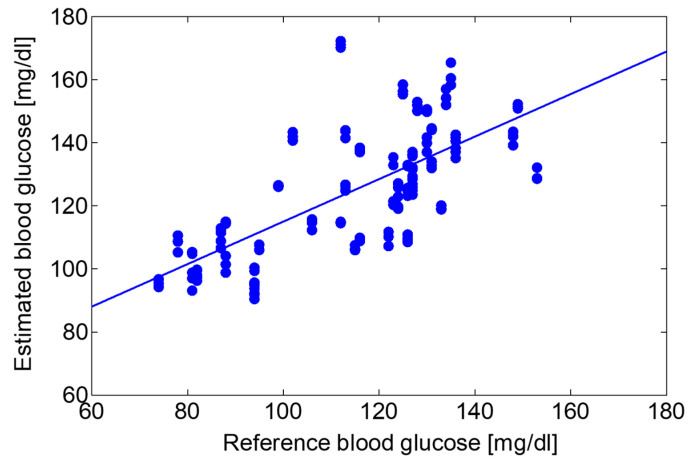
The correlation between the estimated values and the reference blood glucose levels measured by blood sampling.

To test the feasibility of the proposed system for healthcare applications, we applied the partial least squares regression (PLSR) method to the absorption spectra of the human lips to estimate blood glucose levels. In the test, a healthy adult subject measured the optical absorption spectra of the lips after taking a meal every 15 min for roughly 2 h. During the same time, blood glucose levels were measured using a conventional blood glucose self-monitoring device (Johnson & Johnson, New Brunswick, USA, One Touch Verio Vue). In the PLSR analysis, the spectrum data with 4800 data points in the wavelength region of 1050 to 1200 cm^−1^ were analyzed. Figure 10 shows the regression result from data over 4 days (n = 132), and the coefficient of determination R^2^ was 0.71. Then, by using the regression equation obtained in Figure 10, we estimated blood glucose levels from the optical absorption spectra that were not used in the above analysis. In this estimation, the data of the same subject taken over 5 days (n = 150) were used. Figure 11 shows the correlation between the estimated values and the reference blood glucose levels measured by blood sampling.

## 4. Discussion

We newly adopted a high-speed wavelength-swept, pulsed QCL as a light source of the mid-infrared spectroscopic system for healthcare applications. This type of QCL with MEMS-scanning grating has the advantages of compactness and low cost over conventional QCLs because it is advantageous in terms of cooling and downsizing. To obtain continuous spectra from the comb-shaped output spectrum of the pulsed QCL, however relatively complicated processes such as peak detection and data smoothing, were necessary. Then we propose to simply integrate the comb-shaped spectra, the timing of which was slightly shifted. With a spectroscopic system using a conventional HgCdTe detector with an amplifier of 150 kHz bandwidth, continuous spectra were obtained as shown in Figure 4. Although the absorption spectrum of glucose aqueous solution measured using the QCL-based system was in correspondence with that obtained using the conventional FT-IR based system, we found some noises on the spectrum and the lower concentration limit was 3%.

To improve measurement sensitivity, the height of the ATR prism was changed from 2.4 mm to 1.2 mm. Then we observed, as shown in Figure 5, a clear peak at 1080 cm^−1^ on the absorption spectra of glucose solution even at a concentration of 0.5%. This is because the number of reflections at the top and bottom surfaces of the prism was increased from 9 to 19 by changing the prism height from 2.4 mm to 1.2 mm. As the length of the prism was fixed at 24 mm to fit the size of the human lips, the introduction of a thin prism is effective to improve the sensitivity.

In the next step, we newly adopted a high-speed amplifier combined with an InAsSb detector. A narrower pulse width obtained from this amplifier reduces the overlapping of each pulse of the comb shape spectrum and this enables integration of a large number of spectra to reduce noise as shown in Figure 6 and Figure 7. Moreover, as the size of the electronically cooled InAsSb detector (4 × 4 × 7 cm^3^ including the amplifier) is much smaller than that of the liquid N_2_-cooled HgCdTe detector, it is preferable for practical use.

Using the improved system, as shown in Figure 8, we confirmed that the SNR had significantly improved compared with the solution spectrum with a concentration of 1%, measured by the system prior to improvement. Furthermore, absorption peaks could be observed, even in a solution with a concentration as low as 0.1%. Then we measured an absorption spectrum of human lips as shown in Figure 9 and found that the spectrum shape coincided well with the spectrum measured by the FT-IR system, and that clear absorption peaks at 1080, 1120, and 1160 cm^−1^ were observed by the QCL-based system. These peaks observed in the absorption spectra of human lips coincide well with the ones that appeared in the glucose aqueous solution. However, we found that the peak intensities do not correlate well with blood glucose levels.

Using the proposed system, it is expected to investigate the spectrum formed by components such as proteins, sugars, and lipids contained in blood or interstitial fluid non-invasively. As an example, we tried to estimate blood glucose levels from the absorption spectrum of human lips. Continuous spectra obtained using this system enables the application of multivariate analysis that provides more precise investigation compared to simple analyses focusing on peak intensities of a single or multiple absorption peaks. We adopted the PLSR method because usually collinearity exists among the components contained in human tissues. We firstly applied PLSR analysis to the 132 measured spectra and obtained a regression equation providing the determination coefficient of 0.71 as shown in Figure 10. Then we estimated blood glucose levels by applying the equation to the other 150 data. Because a good coefficient of determination of 0.49 was obtained, we showed that it is possible to estimate blood glucose levels in unknown data series.

## 5. Conclusions

We proposed a mid-infrared spectroscopic system using a high-speed wavelength-swept, pulsed QCL for healthcare applications. The QCL with a MEMS-scanning grating enabled a spectroscopic system with the advantages of compactness and low cost over the common EC-QCL-based systems. We built an ATR measurement system comprising the QCL, hollow optical fibers, and an InAsSb detector and tested its measurement sensitivity using aqueous solution samples.

A continuous spectrum was obtained by integrating the comb-shaped spectra, the timing of which was minimally shifted. Since this method did not require complex calculations, absorption spectra were obtained in real-time. We found that the SNR of the obtained spectrum had been improved by increasing the number of spectra that were integrated into the spectrum calculation; as a result, we succeeded in measuring the absorption spectrum of a 0.1% aqueous glucose solution. To test the feasibility of the system for healthcare applications, the absorption spectra of the human lips were measured. It was shown that estimating blood glucose levels is possible using a model equation derived by PLSR analysis of measured absorption spectra.

A spectroscopic system using a wavelength-swept QCL emitting mid-infrared light can be a substitute for the conventional FT-IR based systems. In addition, the pulsed QCL using high-speed operating MEMS grating, which was adopted in this paper, is advantageous in the size and cost over the common wavelength-tunable EC-QCL. Therefore, it is expected to develop a compact and low-cost system for healthcare applications by using the MEMS-based pulsed QCL.
